# Doctor-patient relationship improved during COVID-19 pandemic, but weakness remains

**DOI:** 10.1186/s12875-021-01600-y

**Published:** 2021-12-22

**Authors:** Yanan Zhou, Yuejiao Ma, Winson Fu Zun Yang, Qiuxia Wu, Qianjin Wang, Dongfang Wang, Honghong Ren, Yinli Luo, Dong Yang, Tieqiao Liu, Xiaoming Wu

**Affiliations:** 1 Department of Psychiatry, Hunan Brain Hospital (Hunan Second People’s Hospital), Changsha, China; 2 Department of Psychiatry, National Clinical Research Center for Mental Disorders, The Second Xiangya Hospital of Central South University, Changsha, China; 3 Department of Psychological Sciences, College of Arts & Sciences, Texas Tech University, Lubbock, TX USA; 4 Department of Psychiatry and Psychotherapy, University Hospital Rechts der Isar, Technical University of Munich, Munich, Germany; 5 Department of Cardiovascular Surgery, The Second Xiangya Hospital, Central South University, Changsha, China

**Keywords:** COVID-19, Doctor-patient relationship, PDRQ-9, Importance-performance analysis (IPA), China

## Abstract

**Objective:**

To assess the quality of the doctor-patient relationship (DPR) in China and possible influencing factors during the COVID-19 period from the patient’s perspective.

**Methods:**

An online survey was carried out nationwide from March 12, 2020 to March 30, 2020 in China via a convenience sampling strategy. Patients who met the inclusion criteria were invited to complete a questionnaire regarding the quality of DPR, including sociodemographic information, the Patient-Doctor Relationship Questionnaire (PDRQ-9), and influencing factors for DPR during the pandemic.

**Results:**

A total of 1903 patients were included. Our result showed that participants had a higher PDRQ-9 score during the COVID-19 pandemic (4.18 ± 0.51) than that before the COVID-19 pandemic (3.86 ± 0.67). Importance-performance analysis (IPA) revealed that doctor-patient communication, patient satisfaction, consultation time, doctor’s attitude, and medical knowledge were specific aspects that needed to be prioritized to improve the DPR. Multiple linear regression analysis suggested that positive media reports, telemedicine, and national policies had a significantly positive effect on the DPR during the pandemic (*P* < 0.05).

**Conclusion:**

In general, the DPR had been improved during the COVID-19 pandemic. Our research found the key points that needed to be prioritized to improve the DPR during the pandemic, which may provide effective suggestions for building a harmonious DPR in the future.

**Supplementary Information:**

The online version contains supplementary material available at 10.1186/s12875-021-01600-y.

## Introduction

The doctor-patient relationship (DPR) plays a crucial role in health care, as it is closely associated with treatment adherence, patient satisfaction, and treatment outcome [1–4]. A good DPR is a determinant for patient satisfaction and a better clinical outcome, which affects the management of both chronic and acute disease, regardless of sociocultural factors [5].

It is believed that DPR can be restricted or promoted in different dimensions, i.e., the quality and type, which might affect how both the medical staff and patients view a given medical event [6]. As a special type of social interpersonal relationship shaped and evolved by the environment, DPR is dynamic, and depends on the social and medical situations [7].

During the past year, with the outbreak and protracted course of the COVID-19 pandemic, the healthcare industry has experienced unprecedented challenges. The disease caused by SARS CoV2 soon spread across many countries [8] and caused over-whelming challenges in healthcare service delivery globally in many ways [9–13], including limited resources, appropriate priority setting, availability of medical care, isolation of doctors and patients, information sharing, etc., resulting in disproportional psychological [14] and well-being concerns [15] on both medical staff and patients. Although the pandemic has posed much pressure on the Chinese healthcare system, it also affected how society views medical workers, with many reports referring to medical workers as heroes or “white angels” [16, 17]. Hence, we may infer that the DPR during the pandemic might have been altered accordingly.

Since the outbreak of COVID-19, the DPR has attracted the public attention in China, which was reflected by increased searches of “COVID-19” and “DPR” in Baidu, a leading search engine in China [18]. Yet, it is unclear what impact the pandemic has on DPR in this country. In clinical practice, DPR is usually measured by patients’ perception [19], which was regarded as the feedback of medical service quality [20]. Based on the advantages of comparability and external validity of results, quantitative assessment using a validated scale is the most common method to measure doctor-patient interaction [21]. However, to date, there has been few studies using validated scales to assess DPR during the pandemic in China.

Therefore, in the present study, we aim to investigate the impact of COVID-19 pandemic on DPR. We compared patient’ perceptions of DPR before COVID-19 and during COVID-19 by using the patient-doctor relationship questionnaire (PDRQ-9), an instrument for evaluating DPR from patients’ perspective [22] with excellent reliability and internal consistency [19]. We also aim to examine how patient perceptions of DPR was impacted by multiple contextual factors, such as patient demographic data and changes in healthcare system in response to the pandemic, as well as to identify the key points for improving DPR. Findings of this study may help us to better understand the relationship between doctors and patients, as well as provide suggestions for future medical practice and healthcare policy.

## Methods

### Study design and setting

This cross-sectional, online study was carried out from March 12 to March 30, 2020 in China using convenience sampling methods. We disseminated the study flyer through online communities or social media sites (e.g., WeChat, Weibo, QQ) to enhance our reach to potential interested participants. In addition, we encouraged each enrolled participant to forward our study to their friends, relatives, colleagues, or other potentially eligible individuals. The flyer contained information about the study (including inclusion criteria), three links (versions for medical staff, patients, and general public, respectively) to Questionnaire Star (a professional online survey platform, https://www.wjx.cn), and the contact information of the researchers. Potential interested individuals can participate in the online survey by visiting the corresponding link on the flyer. The first page of link is the study’s ethics approval consent form, which clearly stated the purpose and benefits of the study. Individuals who willing to voluntarily participate after electronic informed consent were invited to complete an anonymous online questionnaire.

### Eligibility criteria

The eligibility criteria for patients in this study were as follows: 1) being 18 years or above, 2) native or fluent Chinese speaker, 3) having seen a doctor during the pandemic (including online consultation), and 4) did not engage in medical work.

### Outcome measures

The primary outcome measure was the patients’ perception of DPR before and during the pandemic, which was quantitatively evaluated using PDRQ-9. The PDRQ-9 is a simple and easy-to-use questionnaire that assesses DPR from the perspective of patients for scientific purposes and in practice to monitor the DPR in medical settings [19]. It has been translated, validated, and used in many countries, including China [23, 24]. This scale consists of 9 questions on a 5-point Likert scale (1 = Strongly disagree to 5 = Strongly agree). In this article, we used the average score and DPR1-DPR9 to represent each item (see Table 2 for details).

Influencing factors shown to be related to DPR from the results of a focus-group discussion, were also included in the online questionnaire. On the afternoon of the 3 March 2020, we conducted a focus-group discussion with 20 available participants (12 patients and 8 medical staff) in the meeting room of Second Xiangya Hospital. To fulfil the principle of diversity, we purposively selected patients and medical staff from different wards. The discussion was guided by two questions to collect and integrate information: (1) How they perceived the DPR during the pandemic? (2) What do they think about factors might influence DPR during the pandemic? The discussion lasted 50 min and the content was recorded in detail and summarized into 10 items upon the mentioned frequency, with the options for response were negative influence, no influence, and positive influence. In this article, we used factors 1 to 10 to represent these influencing factors (See Table 3 for details).

Sociodemographic information was also collected and included gender, age, education, occupation, residency, yearly income, medical insurance, type of hospital visited, frequency of face-to-face doctor visits during the pandemic.

### Importance–performance analysis

Importance-Performance analysis (IPA) is a technique originally developed by Martilla and James to identify management priorities [25] and has been wide application in various sectors such as education [26, 27], tourism [28, 29], and health care [30, 31]. In this study, we adapted a variant of the traditional IPA as proposed by Yavas et al. [32] to identify existing problems and find breakpoint to improve DPR from the patient’s perspective. It is presented as a grid divided into four quadrants with performance and relative performance as the axes. The horizontal axis shows the performance of DPR before the pandemic, and the vertical axis shows the performance of DPR during the pandemic. The four quadrants are as follows: Quadrant I, in the top-right corner,is the “advantage area”; Quadrant II, in the top-left corner,is the “maintenance area”; Quadrant III, in the bottom-left corner,is the “opportunity area”; Quadrant IV, in the bottom-right corner,is the “improvement area” [33]. Of most interest are attributes in quadrants III and IV, which indicating that clinicians and decision makers should devote further resources to improve its performance in future.

### Data quality control

To ensure the quality of data, we conducted quality control for our sample in addition to our inclusion and exclusion criteria to further flag and exclude untrustworthy responses. A detailed description of the data Quality Control has been presented previously [34]. After scrutinizing the initial data(*N* = 2000), we excluded 97 responses (52 were eliminated because of uncompleted data, another 35 were excluded because their responses couldn’t be logically verified by the platform, and 10 were excluded as their completion time was shorter than the required minimum time of 3 min), and finally, 1903 data were included for the final analysis.

### Statistical analysis

The data were analyzed using SPSS software. Paired-sample t test was used to compare the DPR before and during the pandemic. Independent-samples t test or one-way ANOVA were used to compare difference in average total score of PDRQ-9 between participants with different demographic characters. IPA model was adopted to analyze patients’ perception of DPR, identify existing problems, and find specific targets to improve DPR. Pearson correlation method and multiple linear regression analysis were used to examine the relationship between DPR and influencing factors. The statistical significance level was set at *p* < 0.05 (two-sided).

## Results

### Demographic statistics

The study consists of 1903 patients. More than half (60.00%) were female, about three quarter (74.20%) had at least a college degree, more than three quarter (83.60%) lived in city, only about a quarter(28.3%) earn more than 100 K a year, more than three quarter (79.6%) thought the cost of medical care had a moderate impact on their family’s economy, more than half(62.4%) never or occasionally visit the doctor online rather than face-to-face, and about three quarter(72%) visited the doctor at the prefecture-level hospitals and above. See more demographic characteristics details in Table 1.

**Table 1:** Difference in average total score of PDRQ-9 between participants with different demographic characters during the pandemin

Variables	***N*** = 1903	Mean (SD)	***p***-value
Sex (%)			
Female	1142 (60.00)	4.20 (0.51)	0.354
Male	761 (40.00)	4.17 (0.51)	
Age			
≤ 30	629 (33.10)	4.19 (0.55)	0.749
31–40	797 (41.90)	4.18 (0.47)	
41–50	298 (15.70)	4.16 (0.53)	
> 50	179 (9.40)	4.20 (0.52)	
Education (%)			
Below High School	144 (7.60)	4.23 (0.52)	0.617
High School	347 (18.20)	4.19 (0.51)	
College	1181 (62.10)	4.18 (0.50)	
Master’s and above	231 (12.10)	4.15 (0.56)	
Yearly Income (%)			
< 50 k	771 (40.50)	4.19 (0.51)	0.415
50–100 k	593 (31.20)	4.19 (0.50)	
100–200 k	332 (17.40)	4.14 (0.53)	
> 200 k	207 (10.90)	4.19 (0.51)	
Occupation (%)			
Civil servant	87 (4.60)	4.18 (0.56)	0.612
Institution staff (schools, research, military, etc.)	649 (34.10)	4.15 (0.53)	
Medical Student	148 (7.80)	4.18 (0.53)	
Non-medical student	127 (6.70)	4.22 (0.56)	
Others	368 (19.30)	4.18 (0.46)	
Retired	53 (2.80)	4.20 (0.49)	
Self-employed	471 (24.80)	4.22 (0.50)	
Residency (%)			
City	1590 (83.60)	4.19 (0.50)	0.830
Town	95 (5.00)	4.15 (0.43)	
Village	218 (11.50)	4.18 (0.59)	
Medical Expenses (%)			
Very little	89 (4.70)	4.26 (0.55)	0.332
Little	299 (15.70)	4.21 (0.46)	
Average	764 (40.10)	4.17 (0.52)	
more than average	459 (24.10)	4.19 (0.47)	
Huge	292 (15.30)	4.15 (0.59)	
Frequency of face-to-face doctor visits during the pandemic (%)			
Never	270 (14.20)	4.20 (0.56)	0.309
Occasionally (1–2 times)	917 (48.20)	4.18 (0.52)	
Sometimes (3–4 times)	463 (24.30)	4.18 (0.45)	
Often (6–12 times)	210 (11.00)	4.14 (0.57)	
Always (> 12 times)	43 (2.30)	4.31 (0.42)	
Hospital level (%)			
Individual clinics	82 (4.30)	4.18(0.47)	0.344
County	300 (15.80)	4.15(0.54)	
Township	130 (6.80)	4.24(0.47)	
Prefecture	786 (41.30)	4.16(0.53)	
Provincial and ministerial	584 (30.70)	4.19(0.49)	
Private	21 (1.10)	4.15(0.63)	

Note: *SD* standard deviation

### Differences in DPR before and during the pandemic

The results showed that the score of each item and the total average score of the PDRQ-9 before and during the pandemic were at a level of about 4 points, indicating the respondents generally believed that the DPR in China was at a good level. Paired-sample t test revealed significant differences in scores of almost all the items (except DPR7) and the average total score before and during COVID-19 (*P* < 0.05), indicating that the respondents believed that the DPR during the pandemic was better than before the pandemic (Table 2).

**Table 2:** Differences in doctor-patient relationship before and during the pandemic measured by PDRQ-9

Variables	Pre-pandemic	During- pandemic	t	*P*
DPR1	4.20 ± 0.79	4.81 ± 0.50	− 52.878	< 0.001
DPR2	3.44 ± 1.02	3.48 ± 0.96	−6.208	< 0.001
DPR3	4.06 ± 0.82	4.50 ± 0.74	−25.85	< 0.001
DPR4	3.74 ± 0.94	4.43 ± 0.78	−46.379	< 0.001
DPR5	3.88 ± 0.9	4.52 ± 0.72	−43.496	< 0.001
DPR6	3.82 ± 0.88	3.83 ± 0.85	−2.503	0.012
DPR7	4.00 ± 0.84	4.01 ± 0.80	−1.688	0.091
DPR8	3.87 ± 0.88	4.15 ± 0.65	−25.633	< 0.001
DPR9	3.73 ± 0.96	3.92 ± 0.80	−16.342	< 0.001
Average total score	3.86 ± 0.67	4.18 ± 0.51	−52.687	< 0.001

Note: DPR1: My doctor helps me; DPR2: My doctor has enough time for me; DPR3: I trust my doctor; DPR4: My doctor understands me; DPR5: My doctor is dedicated to help me; DPR6: My doctor and I agree about the nature of my medical symptoms; DPR7: I can talk to my doctor; DPR8: I feel content with my doctor’s treatment; DPR9: I find my doctor easily accessible

### Importance-performance analysis (IPA)

**Fig. 1:**
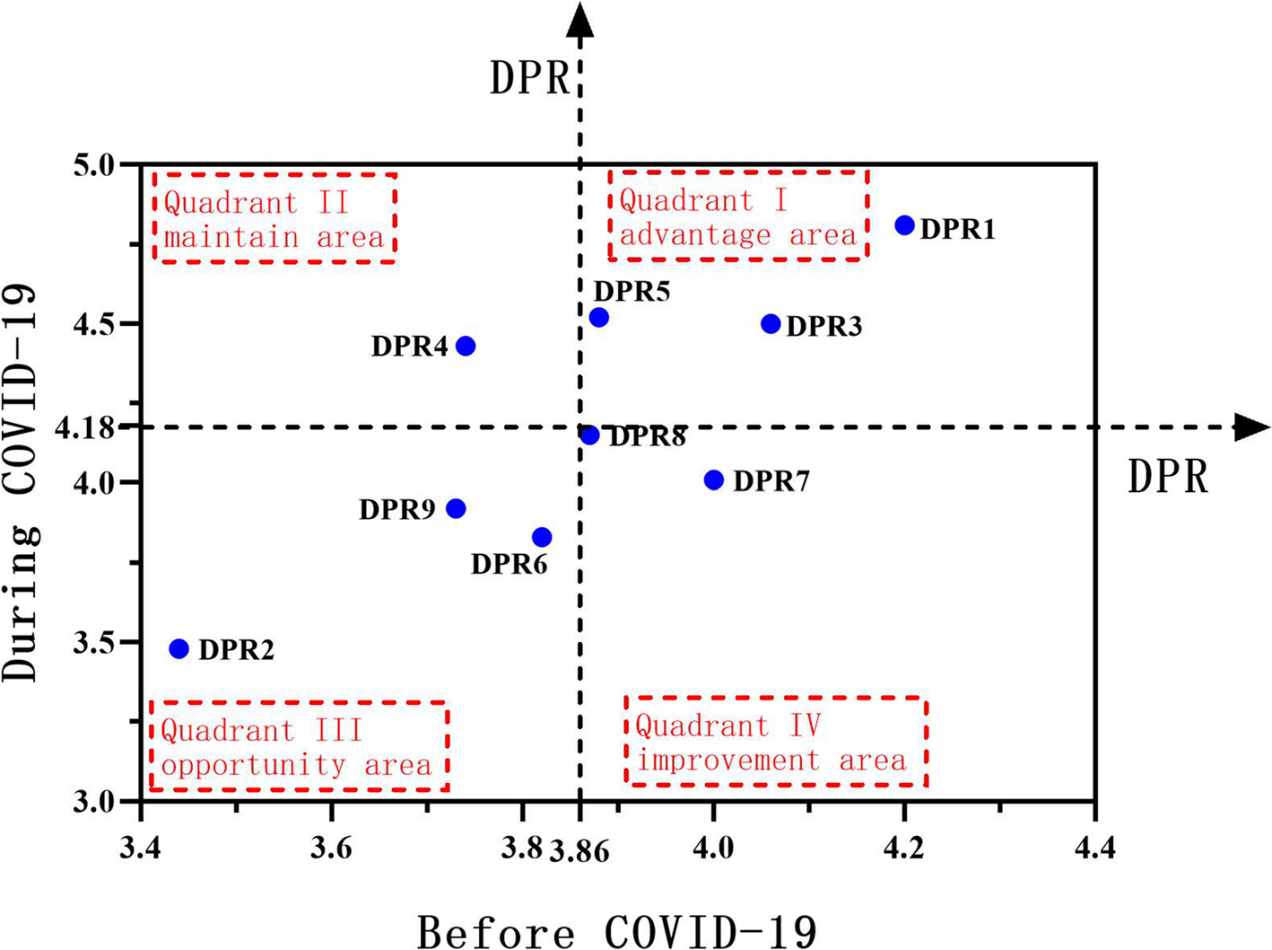
IPA analysis of doctor-patient relationship before and during the pandemic

According to the values obtained in Table 1, the IPA representation was performed in Fig. 1. It can be observed that DPR1, DPR3, and DPR5 fell in Quadrant I (“advantage area”), indicating high quality both before and during the pandemic. DPR4 fell in Quadrant II (“maintain area”), which indicated high score during the pandemic but relatively low score before the pandemic. Therefore, keeping these items of Quadrant I and Quadrant II stable can be beneficial. DPR2, DPR6, and DPR9 fell in Quadrant III (“opportunity area”), indicating low scores both before and during the pandemic. However, it does not mean we can neglect the items in this area; instead, special attention needs to be paid on the cause analysis and breakout points should be identified to improve DPR. DPR7 and DPR8 fell in Quadrant IV (“improvement area”), indicating high scores before the pandemic but low scores during the pandemic; this implies that immediate action was needed to improve the present situation (see Fig. 1).

### Influencing factors of DPR during the pandemic

Independent sample t test and one-way ANOVA revealed that there was no significant difference in average total score of PDRQ-9 between participants with different demographic characters during the pandemic (Table 1).

Pearson correlation analysis showed significantly positive correlation between the mean value of DPR and factors 1, 3, 4, 5, 7, 8, 9, and 10 (Table 3). Further multiple linear regression analysis showed that factor 3 (positive media reports on medical staff), factor 9 (free online consultations, psychological hotlines, and other activities), and factor 10 (free treatment for confirmed and suspected COVID-19 patients) had a significantly positive effect on DPR (*P* < 0.05) (Table 4).

**Table 3:** Correlation between doctor-patient relationship and the 10 influencing factors

	Factor1	Factor2	Factor3	Factor4	Factor5	Factor6	Factor7	Factor8	Factor9	Factor10
PDRQ-9	.073^a^	0.041	.067^a^	.058^b^	.067^a^	0.029	.050^b^	.059^a^	.107^a^	.109^a^

Note: Factor 1: Better understanding of the work of medical staff; Factor 2: Aware of limitations of medicine; Factor 3: Positive media reports on medical staff; Factor 4: Measures to encourage and care for medical professionals; Factor 5: Troublesome and inconvenient process of medical consultation during the pandemic; Factor 6: Disproportionate frontline and insufficient hospital staff; Factor 7: Public’s nervousness and panic during the pandemic; Factor 8: Dissemination of knowledge related to the pandemic; Factor 9: Free online consultations, psychological hotlines, and other activities; Factor 10: Free medical treatment to confirmed and suspected COVID-19 patients

^a^Correlation is significant at the level of 0.01 (2-tailed)

^b^Correlation is significant at the level of 0.05 (2-tailed)

**Table 4:** Multiple linear regression of doctor-patient relationship during the pandemic

	Unstandardized Coefficients	t	***P*** value	95% Confidence Interval	
				Lower Bound	Upper Bound
Factor 1	0.040	1.028	0.304	0.036	0.116
Factor 3	0.032	1.987	0.047^a^	< 0.001	0.063
Factor 4	0.001	0.030	0.976	0.094	0.097
Factor 5	0.023	1.225	0.221	0.014	0.059
Factor 7	−0.034	−0.688	0.492	0.064	0.132
Factor 8	−0.038	−1.163	0.245	0.026	0.102
Factor 9	0.094	2.242	0.025^a^	0.012	0.177
Factor 10	0.106	2.323	0.02^a^	0.017	0.196

Note: Factor 1: Better understanding of the work of medical staff

Factor 3: Positive media reports on medical staff

Factor 4: Measures to encourage and care for medical professionals

Factor 5: Troublesome and inconvenient process of medical consultation during the pandemic

Factor 7: Public’s nervousness and panic during the pandemic

Factor 8: Dissemination of knowledge related to the pandemic

Factor 9: Free online consultations, psychological hotlines, and other activities

Factor 10: Free medical treatment to confirmed and suspected COVID-19 patients

^a^Correlation is significant at the level of 0.05 (2-tailed)

## Discussion

In this study, we compared the quality of DPR before and during the pandemic and explored possible influencing factors that affect DPR during COVID-19 from the perspective of patients. Our findings revealed that respondents were optimistic about the DPR in China and reported an improved DPR during the pandemic. We also found that positive media reports, telemedicine, and medical policies significantly and positively affected the DPR during the pandemic. Furthermore, it was also found that doctor-patient communication, patient satisfaction, consultation time, doctor’s attitude and medical knowledge were specific aspects that needed to be prioritized to improve DPR in the future.

Respondents reported a better DPR in the present study, which was consistent with mainstream media coverage during the outbreak. According to our results, the improvement of DPR is mainly attributed to three factors, i.e., medical policies, positive media reports, and telemedicine. To respond to the COVID-19 pandemic, the Chinese government has taken nationwide and comprehensive measures [35]. One of the main actions taken by the government was free medical observation and treatment for confirmed cases, suspected cases, and close contacts [36]. This action helped form a firm doctor-patient relationship and reduce suspicion and mistrust between both sides. The media also played an important role at different levels in mobilizing public participation, shaping public sentiment, and improving awareness [37]. In the fight against the pandemic, many reports referred to Chinese medical staff as heroes and praised their hard work through media [16, 17]. This enhanced public understanding and support of medical staff, which in turn improved the DPR. Moreover, in the face of COVID-19, telemedicine demonstrated substantial benefits by providing effective consultations, remote patient monitoring, and prevention and treatment guidance for both the public and medical staff without transferring to physical location [38, 39]. During the pandemic in China, a multimodal telemedicine network combing smartphone APPs, 5G services, and existing telemedicine systems was activated immediately [40], and several types of online health services have been provided for people in need [41, 42]. The above measures were proved acceptable, feasible, and effective to improve health care outcomes and DPR in China. In line with our findings, Xu et al. claimed that free medical care, treatment equality, mutual understanding and cooperation, effective and informative communication, positive media reports lead to a harmonious DPR in mobile cabin hospitals during the COVID-19 in China [43]. However, according to a survey of DXY forum, only 13.94% of people believe that the COVID-19 situation could improve the DPR in China [18], which was inconsistent with our findings. A possible explanation for this discrepancy might be differences in participants, methods, and tools. DXY is a medical website with most users being young medical staff, while the participants of our study are patients. In addition, we used PDRQ-9 scale to quantitatively evaluate DPR while the DXY survey used only one question.

In Western studies, there are some different voices in the assessment of DPR during the pandemic. Similar to our study, general practitioners in Italy experienced an improvement of DPR during the early stage of the COVID-19 pandemic in terms of patient understanding, compliance, and solidarity [44]. In the US, many patients expressed positive feelings towards medical service providers and had a better understanding of the evolving field of healthcare facing the challenges [45]. However, Roubille et al. claimed that confidence vanished or impaired with accumulated distrust due to COVID-19 [46]. A possible reason for the inconsistencies is a combination of economic, political, social, and cultural differences. There is a need for cross-cultural and cross-setting studies to explore this complex topic.

Another strength of this study was that the IPA analysis revealed specific priorities for improving the DPR, which were mainly items fell in Quadrant III and Quadrant IV. Items regarding communication and patient satisfaction fell in Quadrant IV (“concentrate here”), indicating that these aspects were in urgent need for improvement and needed attention and intervention from clinicians and policymakers. Effective communication is essential for medical practice and DPR [47], and patient satisfaction may influence treatment compliance, continuity, and communication between doctors and patients [48]. During the pandemic, strict preventive measures such as lock-down, face mask and personal protective equipment were used, which created obstacles for effective doctor-patient communication [49, 50] and posed direct impact on both patients and doctors [50, 51]. Hence, when speaking to patients, doctors need to use patient-centered strategy with clear language [52], as well as eye contact, body gestures and movements [53], to improve patients’ confidence and build a doctor-patient rapport.

Three items fell into Quadrant III (“low priority”). Among them, consultation time ranked last both before and during the pandemic, indicating that the current consultation time was inadequate from the patient’s perspective. Qiao et al. found that shorter consultation time could negatively affect the DPR [24]. Cape and Mohd also found that shorter communication with doctor is a common cause of patient dissatisfaction [54, 55]. In China, the bed-to-nurse ratio is far below the level set by the Ministry of Health, and the workload of medical staff is so heavy that the time allotted to each patient is significantly reduced. Previous study showed that the consultation time in Chinese provincial hospital is only about 3–5 min [24]. Therefore, trying to reduce the workload of doctors and ensure adequate time for consultation will be effective to improve DPR. The doctor’s attitude is also an important factor for DPR. According to some patients, an open and friendly attitude of the doctor makes them feel respected and valued; they also expect close attention and enough time from their doctors [56]. This suggests that doctor’s understanding of doctor-patient communication should not be limited to the length of time but also the attitude and overall quality. It is worth noting that the item 6 (medical knowledge) showed low scores both before and during the pandemic. This might be related to the information asymmetry, which led to misunderstandings and disharmony between doctors and patients [57]. From this perspective, approaches such as medical education [58] and shared decision-making [59] are required to narrow the information gap between doctors and patients.

The present study has important implications for the future of medical services in China. For example, better medical education for both patients and medical professionals can help improve medical services delivery to patients [60], as well as promote public awareness of the current crisis. This may help stabilize public sentiment in the face of uncertainty and maintain social trust [61]. Regular updates of information about COVID-19 and latest policies and services can also promote trust between patients, doctors, and healthcare administrators. Media portrayal of doctors during the pandemic are crucial for the public perception of doctors, which may affect DPR directly. Moreover, DPR can also be affected by other factors such as healthcare system and administration, culture, and financial management medical sectors [62]. With all the above measures, medical services will be improved nationally during the pandemic and the public better prepared for the crises [63].

There are some limitations that should be mentioned. First, due to the nature of the questionnaire and the sampling method, selection bias cannot be ignored. With the use of the convenient online sampling strategy, the population included in this study might not be able to reflect the general population. Second, the measurement of DPR before and during the COVID-19 pandemic were evaluated with the same questionnaire and was based on patients’ self-reports, which may lead to a recall bias. Third, the data disclosed in a previous article might induce over representatives [34]. Four, due to the cultural circle and the specificity of the social relationship between doctors and patients in China, the results of this study cannot be extrapolated to other parts of the world. Therefore, further studies are needed to confirm the impact of COVID-19 on DPR in cross-cultural contexts. As the impacts of medical policies and media on social attitudes still remain unknown, these results should be interpreted with caution. In addition, there are some other factors (such as equal health services and social discrimination) that may affect DPR and need to be verified in follow-up studies.

## Conclusions

In conclusion, this study investigated and compared the doctor-patient relationship before and during COVID-19 from the patient’s perspective. We have identified the main factors leading to better DPR during the pandemic and key points that need to be prioritized for improvement. Our findings may help us better understand the doctor-patient relationship and provide a reference for building a harmonious doctor-patient relationship in the future.

## Supplementary Information

**Additional file 1.**

**Additional file 2.**

## Data Availability

The data is available from the corresponding author on reasonable request and subject to Ethics Board approval.

## Abbreviations

DPR: Doctor-patient relationship
COVID-19: Novel coronavirus disease 2019
PDRQ-9: Patient-doctor relationship questionnaire
IPA: Importance-performance analysis
