# Supplementary material for: Doctor-patient relationship improved during COVID-19 pandemic, but weakness remains
Source: BMC Fam Pract. 2021 Dec 22;22:255. doi: 10.1186/s12875-021-01600-y (PMC8694760; doi:10.1186/s12875-021-01600-y)
Supplement: Supplementary file 1 — Additional file 1. [file 12875_2021_1600_MOESM1_ESM.docx]

1.您的性别：【单选题】

①男

②女



2.您的年龄：【填空题】

3.您的文化程度：【单选题】

①初中及以下

②高中/中专

③大专/本科

④研究生及以上

4.您的个人年收入约为【单选题】：

①≤ 5万

②6-10万

③11-19万

④≥ 20 万

5.您大部分时间居住在：【单选题】

①城市

②乡镇

③农村

6.疫情期间，您居住在：【单选题】

①武汉市

②湖北（武汉以外）

③湖北以外（国内）

④国外

7.本次疫情期间，您属于以下哪种情况：【单选题】

①未感染者

②疑似感染者

③确诊感染者

④不确定

8.您的职业【单选题】

①学生（医学生,尚未参加临床实践）

②学生（非医学生）

③公务员

④国企、事业单位职员、教师、科研人员、军人等

⑤个体经营者/自由职业者

⑥退休

⑦其他

9.您目前的医保类型：【多选题】

①无医保

②城镇职工基本医疗保险

③城镇居民基本医疗保险

④新型农村合作医疗

⑤公费医疗

⑥其他

10. 是否购买商业性医疗保险：【单选题】

①有

②无

11.医疗费用的支出对您家庭经济的影响如何：【单选题】

①非常大

②比较大

③一般

④比较小

⑤非常小

12.疫情期间，您到医院与医生面诊的次数：【单选题】

①未去过

②偶尔（1-2次）

③有时（3-4次）

④经常（6-12次）

⑤总是（> 12次）

13.您最常去哪种医院看病【单选题】

①乡镇级医院

②县级医院

③地市级医院

④省部级医院

⑤私人医院

⑥个体诊所

14.您觉得**近年来中国的**医患关系如何？【单选题】

① 很和谐

② 比较和谐

③ 不知道（没有看法）

④ 比较紧张

⑤ 很紧张

15.您觉得**疫情期间**医患关系如何？【单选题】

① 很和谐

②比较和谐

③ 不知道（没有看法）

④ 比较紧张

⑤ 很紧张

16.与前几年相比，您认为疫情期间的医患关系有何变化？【单选题】

① 明显改善

② 有所改善

③ 无变化

④ 有所恶化

⑤ 明显恶化

17.请分别根据疫情前和疫情期间的就医经历，勾选您对以下说法的认同程度 (各条目得分范围1～5分, 1表示非常不认同 5表示非常认同)。【矩阵题】

|  | **疫情前** | | | | | **疫情期间** | | | | |
| --- | --- | --- | --- | --- | --- | --- | --- | --- | --- | --- |
|  | 1 | 2 | 3 | 4 | 5 | 1 | 2 | 3 | 4 | 5 |
| 我认为医生对我有所帮助 |  |  |  |  |  |  |  |  |  |  |
| 医生有足够的时间给我看病 |  |  |  |  |  |  |  |  |  |  |
| 我信任医生 |  |  |  |  |  |  |  |  |  |  |
| 我觉得医生能理解我 |  |  |  |  |  |  |  |  |  |  |
| 我觉得医生非常愿意帮助我 |  |  |  |  |  |  |  |  |  |  |
| 我和医生对于疾病的看法一致 |  |  |  |  |  |  |  |  |  |  |
| 我可以和医生良好沟通 |  |  |  |  |  |  |  |  |  |  |
| 我对医生给我的治疗很满意 |  |  |  |  |  |  |  |  |  |  |
| 我感觉医生平易近人 |  |  |  |  |  |  |  |  |  |  |

18.以下问题反映您对与您接触医生的信任程度，请选出您对以下说法的认同程度(各条目得分范围1～5分, 1表示非常不认同 5表示非常认同)。【矩阵题】

|  | 1 | 2 | 3 | 4 | 5 |
| --- | --- | --- | --- | --- | --- |
| 为了保证我的健康，医生会做所有能做的事情 |  |  |  |  |  |
| 医生总是以是否方便作为治疗选择的依据，而不会考虑这些治疗是否适合我 |  |  |  |  |  |
| 医生的水平没达到我认为医生应该达到的程度 |  |  |  |  |  |
| 医生很细致、很体贴 |  |  |  |  |  |
| 我觉得医生选择的治疗方案对我是最合适的 |  |  |  |  |  |
| 医生会向我解释可能采取的各种治疗方案的利弊 |  |  |  |  |  |
| 我觉得医生没有认真倾听我向他反映的情况 |  |  |  |  |  |
| 医生把我的利益放在第一位，而不是他自己或医院的利益 |  |  |  |  |  |
| 我可以毫不犹豫的将我的生命交给医生 |  |  |  |  |  |
| 总之，我信任医生 |  |  |  |  |  |

19.您认为以下因素对疫情期间的医患关系有何影响？【矩阵题】

|  | **负面影响** | **无**  **影响** | **正面影响** |
| --- | --- | --- | --- |
| 通过疫情，公众对医务人员的工作有了更多的理解 |  |  |  |
| 通过疫情，公众更认识到医学并不能治愈所有疾病 |  |  |  |
| 媒体对医务人员的正面报道，如医务人员驰援武汉、战斗在抗疫一线等事迹 |  |  |  |
| 国家出台多项措施，激励关爱一线抗疫医务人员 |  |  |  |
| 疫情期间去医院看病的流程更麻烦、更不方便 |  |  |  |
| 医务人员支援抗疫一线，医院人员相对不足 |  |  |  |
| 疫情下人们紧张、恐慌的情绪状况 |  |  |  |
| 疫情期间各学术团体、医疗机构发布关于疫情的相关知识 |  |  |  |
| 疫情期间各学术团体、医疗机构在线提供义诊、心理热线、免费咨询等活动 |  |  |  |
| 国家出台政策对新冠肺炎确诊和疑似患者免费救治 |  |  |  |

20.请根据您的实际情况，选出您对以下说法的认同程度。(各条目得分范围1～5分, 1表示非常不信任/尊重 5表示非常信任/尊重)。

|  | **疫情前** | | | | | **疫情期间** | | | | |
| --- | --- | --- | --- | --- | --- | --- | --- | --- | --- | --- |
|  | 1 | 2 | 3 | 4 | 5 | 1 | 2 | 3 | 4 | 5 |
| 本人对医务工作者的尊重程度 |  |  |  |  |  |  |  |  |  |  |
| 其他大多数人对医务工作者的尊重程度 |  |  |  |  |  |  |  |  |  |  |
| 本人对医务工作者的信任程度 |  |  |  |  |  |  |  |  |  |  |
| 其他大多数人对医务工作者的信任程度 |  |  |  |  |  |  |  |  |  |  |

21.请根据实际情况进行选择

|  | **疫情前** | | **疫情期间** | |
| --- | --- | --- | --- | --- |
|  | 有 | 无 | 有 | 无 |
| 您有没有对医护人员进行过言语攻击？ |  |  |  |  |
| 您有没有对医护人员进行过躯体攻击？ |  |  |  |  |
| 您有没有目睹过他人对医护人员进行过言语攻击？ |  |  |  |  |
| 您有没有目睹过他人对医护人员进行过躯体攻击？ |  |  |  |  |

22.您认为疫情之后，医患关系短期内有何变化？【单选题】

① 明显恶化

②稍有恶化

③ 无变化

④ 稍有改善

⑤ 明显改善

23.您认为疫情之后，医患关系长期内有何变化？【单选题】

① 明显恶化

②稍有恶化

③ 无变化

④ 稍有改善

⑤ 明显改善

24.您认为影响医患关系的重要的原因有哪些？【限选5项】

①各种原因导致医患之间信任度低

②看病难、看病贵

③公众对疾病相关知识的缺乏，不了解疾病的诊治过程及预后特征

④患者对医生期望过高，以为医生是万能的

⑤少数医生收红包、回扣，影响了医生的整体形象

⑥媒体对医、药界的某些负面或不真实的报道

⑦正规渠道解决医疗纠纷困难，医闹犯罪成本低，少数人利用医闹谋取利益

⑧医疗技术水平和服务质量不高

⑨医患沟通问题（医师太忙或沟通不到位等）

⑩医院管理不到位，出现医疗纠纷时处理不及时

11医保报销比例低

12.其他，请说明：_______

25.您认为以下哪些措施对改善医患关系最重要【限选5项】

①广泛宣传医学科普知识，让大众理解医学不是万能的

②提高医疗技术、改善服务质量

③增进医患沟通，如减轻医护工作强度，有更多的时间服务患者

④提升媒体责任担当，加强医疗正面报道，杜绝假新闻

⑤完善医患纠纷的合法处理方式，促进立法、严打恶意医闹

⑥完善医疗保障体制，提高医保覆盖范围

⑦完善医院管理，加强医德医风建设， 树立良好医务人员形象

⑧其他，请说明
