# Supplementary material for: Doctor-patient relationship improved during COVID-19 pandemic, but weakness remains
Source: BMC Fam Pract. 2021 Dec 22;22:255. doi: 10.1186/s12875-021-01600-y (PMC8694760; doi:10.1186/s12875-021-01600-y)
Supplement: Supplementary file 2 — Additional file 2. [file 12875_2021_1600_MOESM2_ESM.docx]

1.your gender：

①male

②female

2.your age：

3.your education level：

①Below High School

②High School

③College

④Master's and above

4.your yearly income:

①<50k

②50-100k

③100-200k

④≥ >200k

5.Where you live most of the time：

①City

②Town

③Village

6.During the outbreak, you lived in：

①Wuhan City

②Hubei (outside Wuhan).

③Outside Hubei (China)

④aboard

7.Which of the following are you in this outbreak:

①Uninfected

②suspected infected

③Confirmed infected

④uncertain

1. Your occupation:

①students (medical students, have not participated in clinical practice)

② students (non-medical students)

③civil servant

④Institution staff (schools, research, military, etc.)

⑤Self-employed

⑥Retired

⑦Others

1. Your current type of medical insurance:：

①no medical insurance

②basic medical insurance for urban employees

③basic medical insurance for urban residents

④New type of rural cooperative medical care

⑤free medical care

⑥others

10. Whether to buy commercial medical treatment insurance：

①Yes

②No

11.How does the cost of medical care affect your family financially:

①Huge

②more than average

③Average

④Little

⑤Very little

1. Frequency of face-to-face doctor visits during the pandemic

①Never

②Occasionally (1-2 times)

③Sometimes (3-4 times)

④Often (6-12 times)

⑤Always (>12 times)

13.The type of hospital you visit most often is:

①County

②Township

③Prefecture

④Provincial and ministerial

⑤Private

⑥Individual clinics

14.What do you think of the doctor-patient relationship in China in recent years?

① very harmonious

② harmonious

③ Don't know (no opinion)

④ tense

⑤ very tense

15.What do you think of the doctor-patient relationship in China during the COVID-19 outbreak?

① very harmonious

② harmonious

③ Don't know (no opinion)

④ tense

⑤ very tense

16.How do you think the doctor-patient relationship has changed during the pandemic compared to previous years

① Significant improvement

② some improvement

③ no change

④ some deterioration

⑤ significant deterioration

1. Please check how much you agree with the following statements according to your medical experience before and during the epidemic (the score range for each item ranges from 1 to 5, 1 means strongly disagree, 5 means strongly agree).

|  | Before covid-19 | | | | | During covid-19 | | | | |
| --- | --- | --- | --- | --- | --- | --- | --- | --- | --- | --- |
|  | 1 | 2 | 3 | 4 | 5 | 1 | 2 | 3 | 4 | 5 |
| My doctor helps me |  |  |  |  |  |  |  |  |  |  |
| My doctor has enough time for me |  |  |  |  |  |  |  |  |  |  |
| I trust my doctor |  |  |  |  |  |  |  |  |  |  |
| My doctor understands me |  |  |  |  |  |  |  |  |  |  |
| My doctor is dedicated to help me |  |  |  |  |  |  |  |  |  |  |
| My doctor and I agree about the nature of my medical symptoms |  |  |  |  |  |  |  |  |  |  |
| I can talk to my doctor |  |  |  |  |  |  |  |  |  |  |
| I feel content with my doctor’s treatment |  |  |  |  |  |  |  |  |  |  |
| I find my doctor easily accessible |  |  |  |  |  |  |  |  |  |  |

1. The following questions reflect your degree of trust in the doctors you contact. Please select your degree of agreement with the following statements (the score of each item ranges from 1 to 5, 1 means strongly disagree, 5 means strongly agree).

|  | Before covid-19 | | | | | During covid-19 | | | | |
| --- | --- | --- | --- | --- | --- | --- | --- | --- | --- | --- |
|  | 1 | 2 | 3 | 4 | 5 | 1 | 2 | 3 | 4 | 5 |
| For sake of my health, my doctor will do whatever I need |  |  |  |  |  |  |  |  |  |  |
| My doctor] always cares more about what is convenient for [him/her] than about my medical needs |  |  |  |  |  |  |  |  |  |  |
| My doctor’s competence level does not achieve the degree I expected) |  |  |  |  |  |  |  |  |  |  |
| My doctor is extremely thorough and careful |  |  |  |  |  |  |  |  |  |  |
| I think my doctor’s treatment decisions are best for me |  |  |  |  |  |  |  |  |  |  |
| My doctor will explain honestly to me about the different treatment options available for me currently |  |  |  |  |  |  |  |  |  |  |
| Sometimes my doctor does not pay full attention to what I am trying to tell [him/her] |  |  |  |  |  |  |  |  |  |  |
| My doctor will act in my interests, not in his/her or the hospital’s interests |  |  |  |  |  |  |  |  |  |  |
| I don’t hesitate to put my life to my doctor’s hands |  |  |  |  |  |  |  |  |  |  |
| All in all, I have complete trust in my doctor |  |  |  |  |  |  |  |  |  |  |

1. How do you think the following factors affect the doctor-patient relationship during the outbreak?

|  | negative  influence | No  influence | positive influence |
| --- | --- | --- | --- |
| Better understanding of the work of medical staff |  |  |  |
| Aware of limitations of medicine |  |  |  |
| Positive media reports on medical staff |  |  |  |
| Measures to encourage and care for medical professionals |  |  |  |
| Troublesome and inconvenient process of medical consultation during the pandemic |  |  |  |
| Disproportionate frontline and insufficient hospital staff |  |  |  |
| Public’s nervousness and panic during the pandemic |  |  |  |
| Dissemination of knowledge related to the pandemic |  |  |  |
| Free online consultations, psychological hotlines, and other activities |  |  |  |
| Free medical treatment to confirmed and suspected COVID-19 patients. |  |  |  |

20. Depending on your situation, choose how much you agree with the following statement. (Each entry has a score range of 1 to 5 points, 1 indicates very distrust/respect 5 indicates very trust/respect).

|  | Before covid-19 | | | | | During covid-19 | | | | |
| --- | --- | --- | --- | --- | --- | --- | --- | --- | --- | --- |
|  | 1 | 2 | 3 | 4 | 5 | 1 | 2 | 3 | 4 | 5 |
| Your personal respect for the medical staff |  |  |  |  |  |  |  |  |  |  |
| The level of respect that most people have for medical staff |  |  |  |  |  |  |  |  |  |  |
| Your personal trust for the medical staff |  |  |  |  |  |  |  |  |  |  |
| The level of trusr that most people have for medical staff |  |  |  |  |  |  |  |  |  |  |

21. Please make a choice based on the actual situation:

|  | Before covid-19 | | During covid-19 | |
| --- | --- | --- | --- | --- |
|  | Yes | No | Yes | No |
| Have you ever verbally assaulted a health care provider? |  |  |  |  |
| Have you ever physically assaulted a health care provider? |  |  |  |  |
| Have you ever witnessed someone verbally assault a health care provider? |  |  |  |  |
| Have you ever witnessed someone physically assault a health care provider? |  |  |  |  |

22. What do you think will happen to the doctor-patient relationship in the short term after the outbreak?？

① Significant improvement

② some improvement

③ no change

④ some deterioration

⑤ significant deterioration

23. 22. What do you think will happen to the doctor-patient relationship in the long term after the outbreak?？

① Significant improvement

② some improvement

③ no change

④ some deterioration

⑤ significant deterioration

24. What do you think are the important factors affecting the doctor-patient relationship? (Choose five)

①Various reasons lead to low trust between doctors and patients

②Medical treatment is difficult and expensive

③The lack of public knowledge about disease

④Patients expect too much from doctors to think they are lmsy

⑤A small number of doctors receive red envelopes, rebates, affecting the overall image of doctors

⑥ Negative or untrue media coverage of the pharmaceutical industry

⑦ Formal channels to resolve medical disputes difficult, low cost of medical crime, a few people use medical trouble for profit

⑧ The level of medical technology and the quality of service are not high

⑨ Doctor-patient communication problems (doctors too busy or poor communication, etc.)

⑩ Hospital management is not in place and medical disputes are not handled in time

(11) The reimbursement rate of medical insurance is low

(12).others，please state：_______

25. Which of the following measures do you think are most important for improving doctor-patient relationship? (Select 5)

①Popularize medical science knowledge widely, let the public understand that medicine is not omnipotent

②Improve medical technology and service quality

③Improved doctor-patient communication, such as less intensive care and more time for patients

④Enhance media responsibility, strengthen positive medical coverage, and eliminate fake news

⑤improve the legal handling of doctor-patient disputes, and promote legislation to crack down on malevolent medical disputes

⑥ improve the medical security system and increase the coverage of medical insurance

⑦ Improve hospital management, strengthen the construction of medical ethics, establish a good image of medical staff

⑧others，please state：_______
